# Supplementary material for: Wildfire precursors show complementary predictability in different timescales
Source: Nat Commun. 2023 Oct 26;14:6829. doi: 10.1038/s41467-023-42597-5 (PMC10603132; doi:10.1038/s41467-023-42597-5)
Supplement: Supplementary file 1 — Supplementary Information [file 41467_2023_42597_MOESM1_ESM.pdf]

Supplementary Information

Wildfire Precursors Show Complementary Predictability in Different Timescales

Yuquan Qu[1*], Diego G. Miralles[2], Sander Veraverbeke[3], Harry Vereecken[1], Carsten Montzka[1]

[1] Institute of Bio- and Geosciences: Agrosphere (IBG-3), Forschungszentrum Jülich GmbH, Jülich, Germany.

[2]Hydro-Climate Extremes Lab, Ghent University, Ghent, Belgium.

[3]Faculty of Science, Vrije Universiteit Amsterdam, Amsterdam, Netherlands.

Supplementary Table

Supplementary Table 1. **All the indices involved in the causal analysis.** The indices marked with '*' were removed from this study according to the data selection result.

Group/Class	Index/Precursor	Calculated?	Product	Spatial Scale	Temporal Scale
Fire	Burned Area (BA)	-	Fire_CCI	250 m	Monthly
Top-down	Potential Evaporation (ET0)	-	ERA5	0.25 degree	Daily
	Aridity Anomaly Index (AAI)	√	(ERA5)	0.25 degree	Daily
	2m Maximum Air Temperature (Tmax)	-	ERA5	0.25 degree	Daily
	Surface Net Solar Radiation (Rad)*	-	ERA5	0.25 degree	Daily
	Vapor Pressure Deficit (VPD)	√	(ERA5)	0.25 degree	Daily
	Total Precipitation (Prec)*	-	ERA5	0.25 degree	Daily
	10m Wind Speed (Wind)	-	ERA5	0.25 degree	Daily
Bottom-up	Soil Water Deficit Index (SWDI)	√	(ERA5)	0.25 degree	Daily
	Fraction of Photosynthetically Active Radiation (FPAR)	-	MCD15A3H	500 m	4-day
	Normalized Difference Vegetation Index (NDVI)	-	MOD13C1	0.05 degree	16-day
	Enhanced Vegetation Index (EVI)	-	MOD13C1	0.05 degree	16-day
	Gross Primary Production (GPP)	-	MOD17A2HGF	500 m	8-day

Supplementary Methods

Precursor Calculations

Aridity Anomaly Index (AAI)

AAI was developed by the Indian Meteorological Department. It is a real-time drought index that describes vegetation water stress. In this study, AAI was calculated by dividing the difference between actual evaporation and potential evaporation by potential evaporation, the formula is shown below. The potential evaporation and actual evaporation are both from ERA5.

$$\mathrm{AAI} = 100 \times \frac{\mathrm{ET_a} - \mathrm{ET_0}}{\mathrm{ET_0}} \qquad \text{Supplementary Equation (1)}$$

where $\mathrm{ET_a}$ is actual evaporation and $\mathrm{ET_0}$ is potential evaporation.

Vapor Pressure Deficit (VPD)

VPD indicates the ability of the atmosphere to extract water from the environment by measuring the difference between the amount of water vapor in the air and how much water vapor it can hold when the air is saturated. In this study, VPD was calculated using minimum and maximum 2m air temperature and dewpoint temperature from ERA5. The formulas are shown below.

$$\mathrm{VPD} = \mathrm{e_s} - \mathrm{e_a} \qquad \text{Supplementary Equation (2)}$$

$$\mathrm{e_s} = \frac{(\mathrm{e_{tmax}} + \mathrm{e_{tmin}})}{2} \qquad \text{Supplementary Equation (3)}$$

$$\mathrm{e_a} = 0.6108 \times \exp\left(\frac{17.27 \times \mathrm{T_{dew}}}{\mathrm{T_{dew}} + 237.3}\right) \qquad \text{Supplementary Equation (4)}$$

$$\mathrm{e_{tmax}} = 0.6108 \times \exp\left(\frac{17.27 \times \mathrm{T_{max}}}{\mathrm{T_{max}} + 237.3}\right) \qquad \text{Supplementary Equation (5)}$$

$$\mathrm{e_{tmin}} = 0.6108 \times \exp\left(\frac{17.27 \times \mathrm{T_{min}}}{\mathrm{T_{min}} + 237.3}\right) \qquad \text{Supplementary Equation (6)}$$

where $\mathrm{e_s}$ is saturation vapor pressure and $\mathrm{e_a}$ is actual vapor pressure. $\mathrm{T_{dew}}$ is dewpoint temperature, $\mathrm{T_{max}}$ is maximum air temperature, and $\mathrm{T_{min}}$ is minimum air temperature.

Soil Water Deficit Index (SWDI)

SWDI is an agricultural drought monitoring index that uses soil moisture (SM), field capacity (FC), and wilting point (WP). The formula can be found below. Soil moisture used in this study is from ERA5 (volumetric soil water layer 1). Field capacity and wilting point are from Montzka et al., 2017[1].

$$\mathrm{SWDI} = 10 \times \frac{\mathrm{SM} - \mathrm{FC}}{\mathrm{FC} - \mathrm{WP}} \qquad \text{Supplementary Equation (7)}$$

Data Selection

Benefiting from the big data era (using remote sensing and reanalysis data), we can now perform a comprehensive wildfire causation study. We started by collecting all the data listed in Table S1. To make the comparison between the top-down and bottom-

up groups more objective and transparent, we used a variable clustering method ("varclushi" package in Python) to execute the precursor selection.

Variable clustering is based on the Principal Component Analysis (PCA) algorithm. Firstly, all the input variables are started in one cluster, and a PCA is done for all the variables in this cluster. Secondly, the Eigenvalues (variance explained by each PC for all the variables) of the principal components are checked. The cluster is split if the second Eigenvalue is larger than a specified threshold (usually set as 0.7 or 1). This cluster-splitting process is iterated until all the second Eigenvalues in all clusters are lower than the specified threshold and the clusters cannot be further split. Thirdly, in each cluster, the representative variables having the highest correlation with their own cluster and the lowest correlation with other clusters are selected according to the formula below:

$$\mathrm{Ratio} = \frac{1 - \mathrm{R}^2_{\mathrm{own}}}{1 - \mathrm{R}^2_{\mathrm{nearest}}} \qquad \text{Supplementary Equation (8)}$$

where $\mathrm{R}^2_{\mathrm{own}}$ is the coefficient of determination (R^2) of the variable with its own cluster, and the $\mathrm{R}^2_{\mathrm{nearest}}$ is the R^2 of the variable with the nearest cluster. The variable with the lowest Ratio is selected in each cluster.

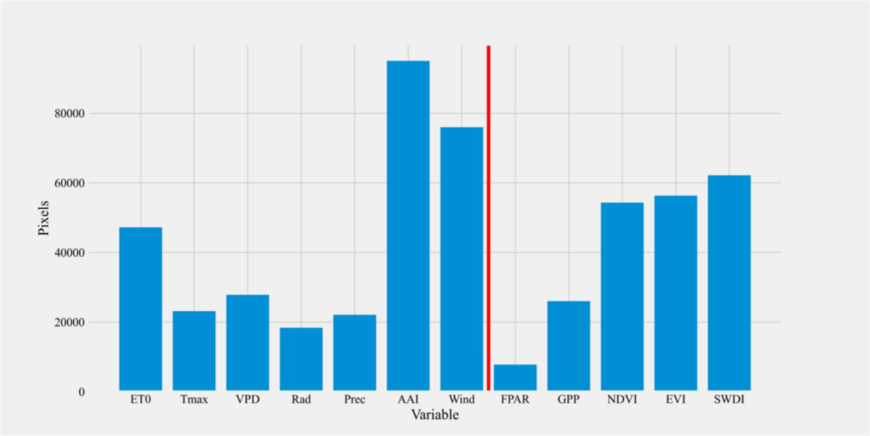

Supplementary Fig 0. Variable clustering result.

We used partial correlation in Peter & Clark Momentary Conditional Independence (PCMCI) to detect casual relationships. Since partial correlation can eliminate the interactions between variables, all variables can be included in the PCMCI analysis. Considering that there are only five bottom-up precursors while seven top-down precursors, we decided to select the top five variables from the top-down group to prevent any artificial inflation of the importance of top-down variables. We applied variable clustering for all 232 ecoregions and recorded the representative variables in each ecoregion. For each variable, we counted globally the pixels where it is representative, the result is shown in Fig S1. According to the number of representative pixels, the variables that were finally selected were AAI, Wind, ET0, VPD, and Tmax in the top-down group and SWDI, EVI, NDVI, GPP, and FPAR in the bottom-up group.

Anomaly Calculation

$$\mathrm{Anomaly} = \frac{\mathrm{y} - \mathrm{mean}}{\mathrm{std}} \qquad \text{Supplementary Equation (9)}$$

where y is the value of a time series at a given time point; mean is the mean of all values at the same time point in all seasonal cycles, in our case, it is the mean of all values for the same week in 18 years (2003–2020); std is the standard deviation derived from all values for the same week of the year across the 18 years.

Supplementary Figures

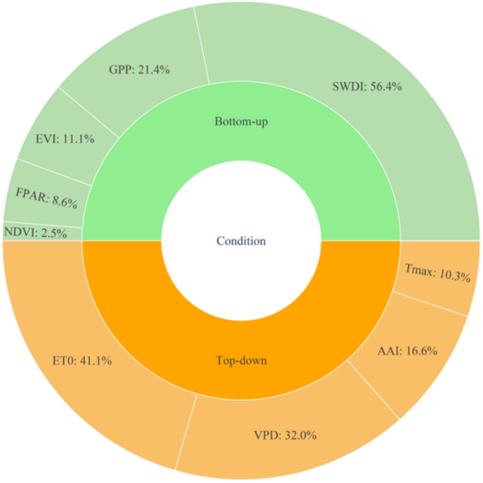

Supplementary Fig 1. Dominant fraction of precursors in each group. The fractions are calculated by dividing the dominant pixel number of each precursor by the dominant pixel number of its belonging group.

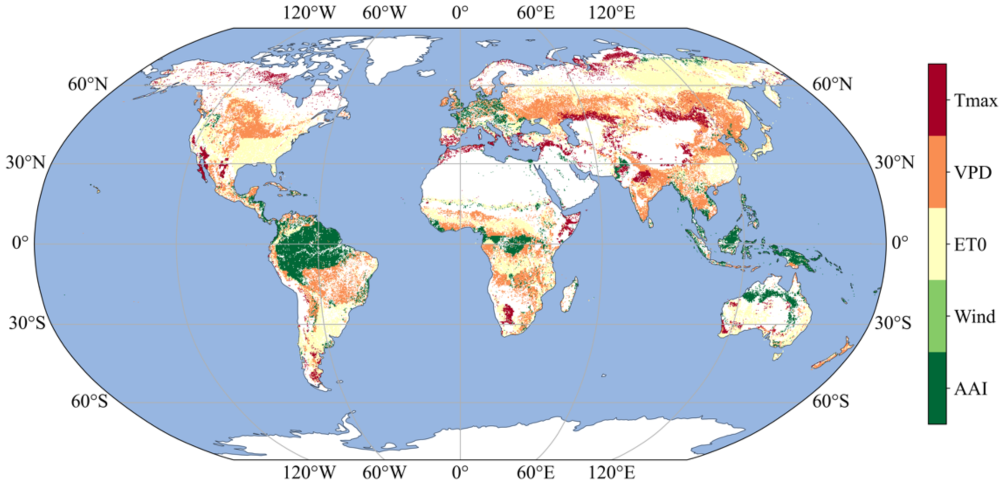

Supplementary Fig 2. Dominant precursors of the top-down group.

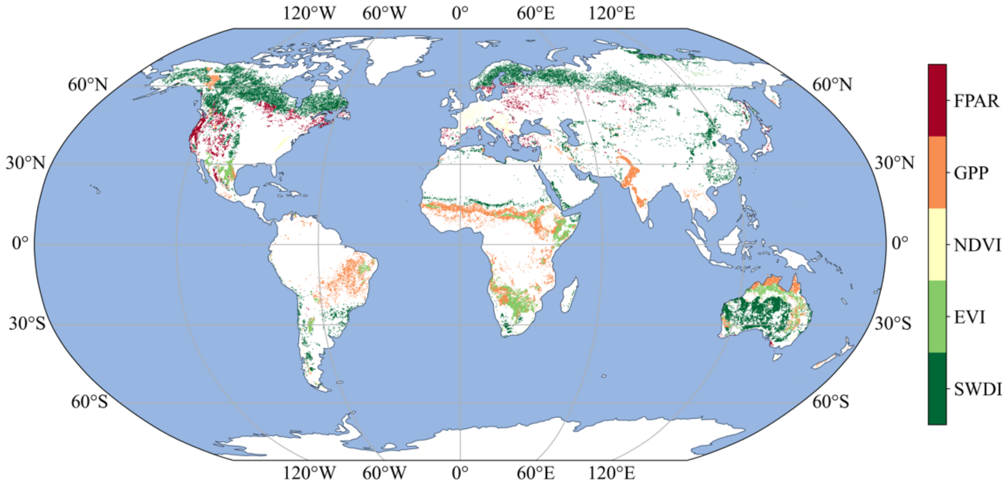

Supplementary Fig 3. Dominant precursors of the bottom-up group.

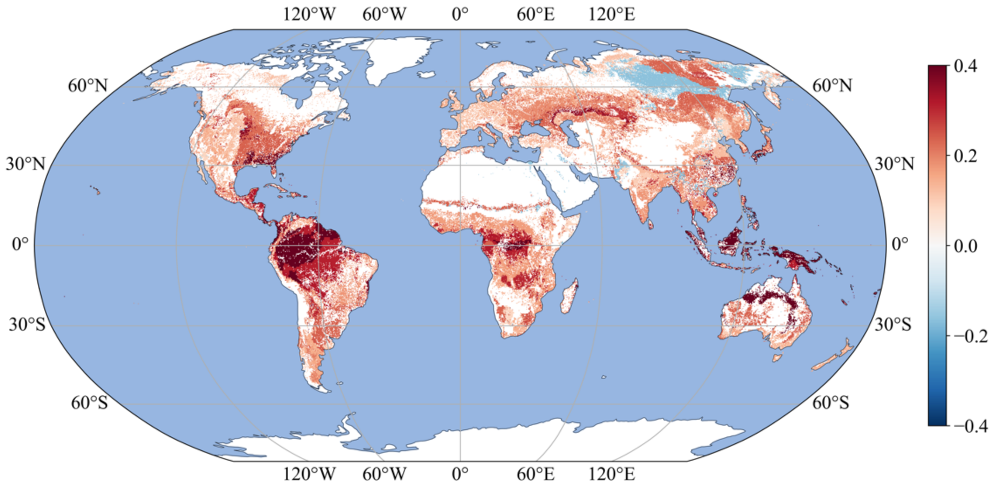

Supplementary Fig 4. **Dominant partial correlations of the top-down group.**

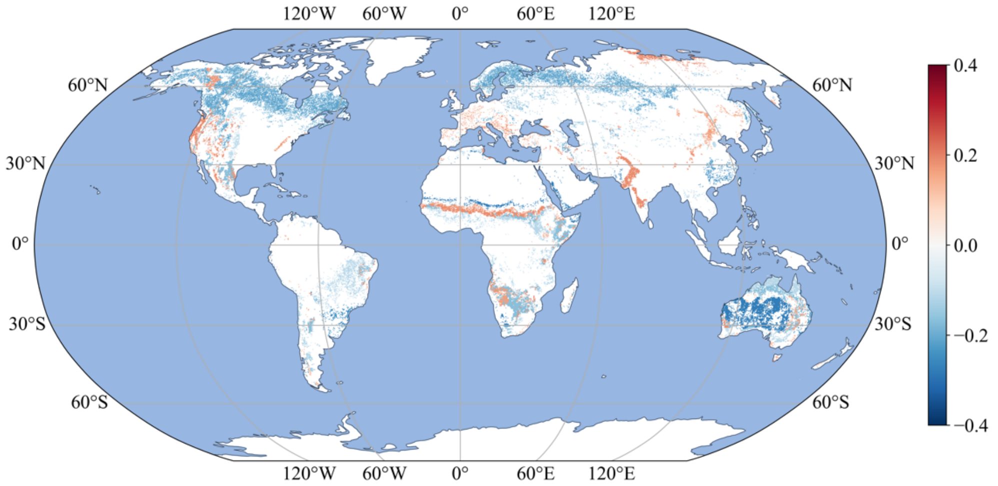

Supplementary Fig 5. **Dominant partial correlations of the bottom-up group.**

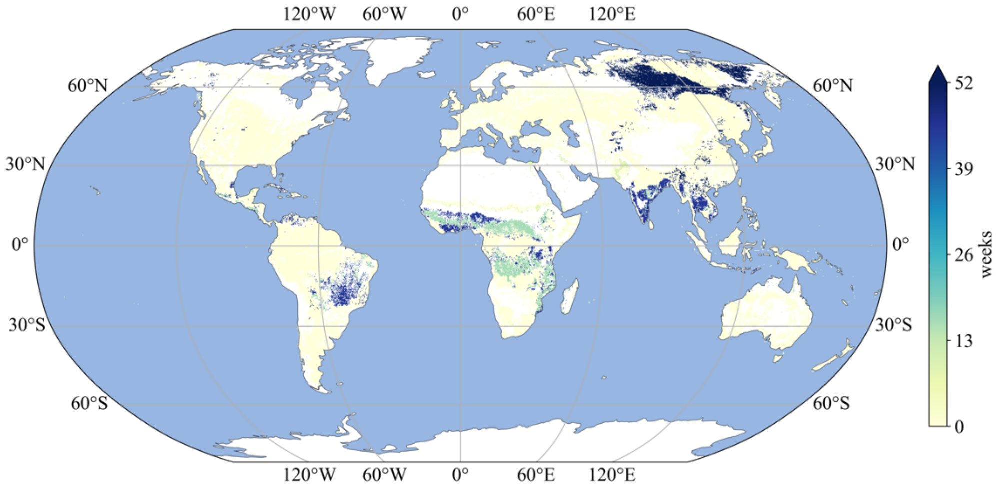

Supplementary Fig 6. **Dominant time lags of the top-down group.**

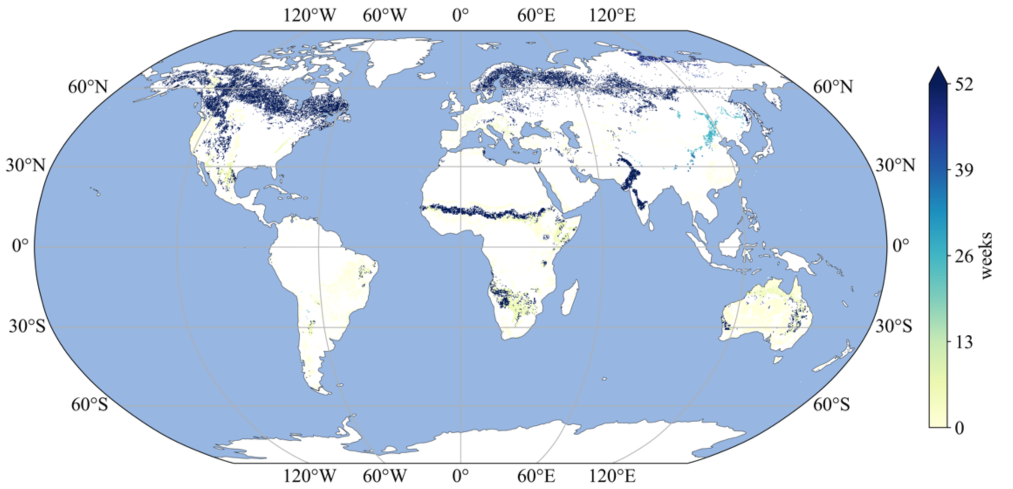

Supplementary Fig 7. **Dominant time lags of the bottom-up group**.

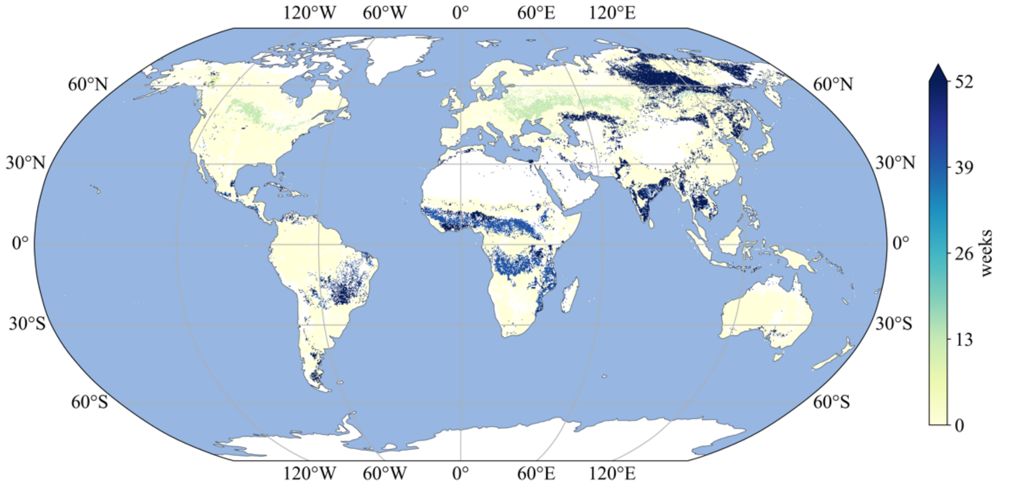

Supplementary Fig 8. **Weighted mean time lags of the top-down group**.

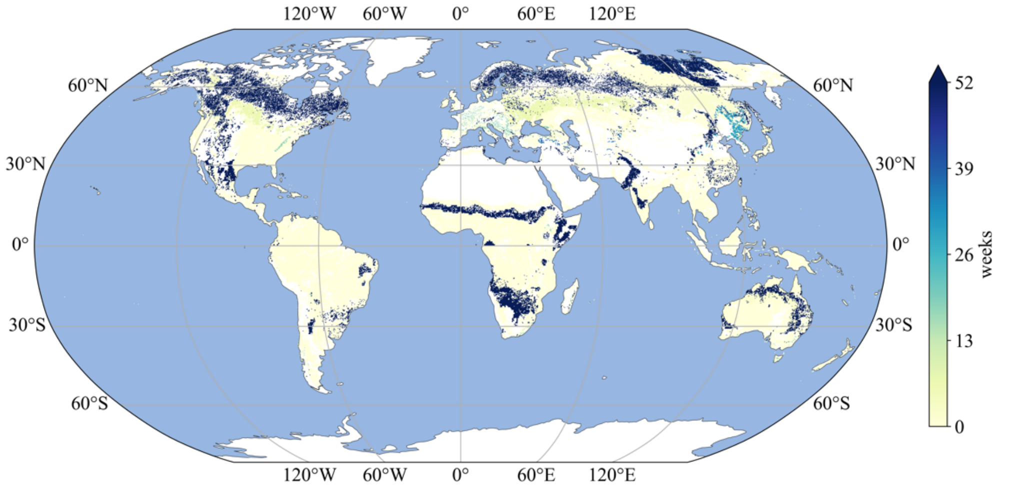

Supplementary Fig 9. **Weighted mean time lags of the bottom-up group**.

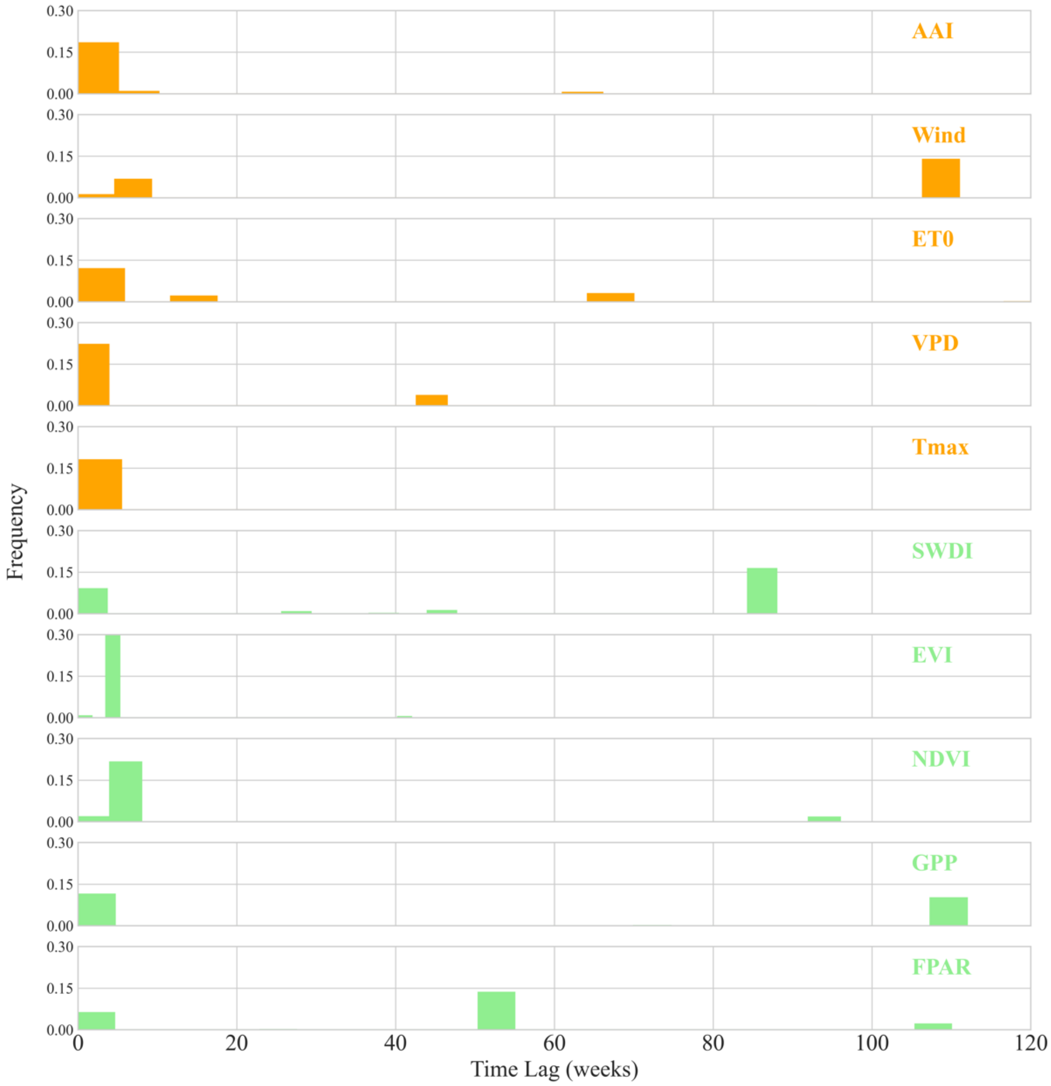

Supplementary Fig 10. The time lag distribution of precursors. For each precursor, only the time lags when one precursor is dominant are used.

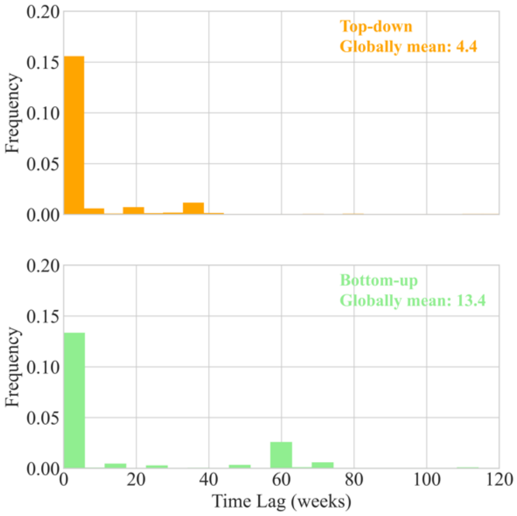

Supplementary Fig 11. The weighted mean time lag distribution of the precursor groups. For each group, only the time lags when this precursor group is dominant are shown.

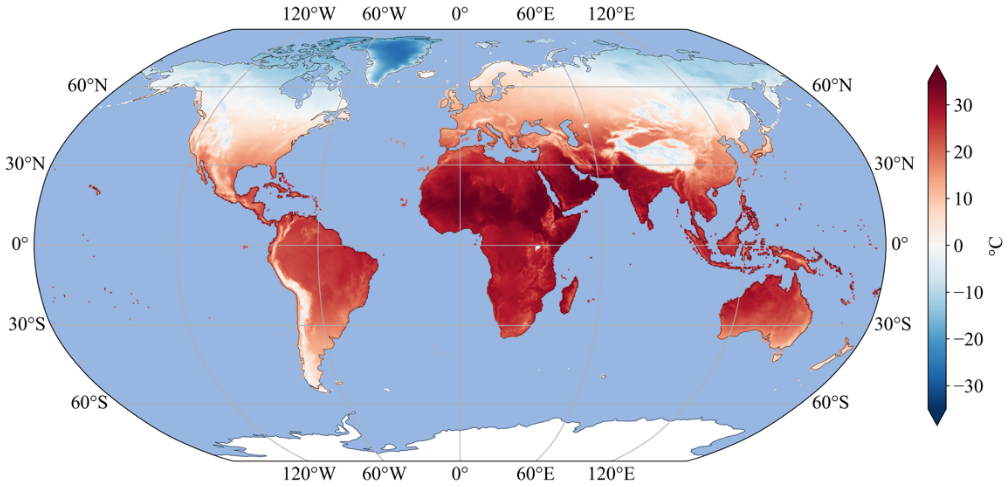

Supplementary Fig 12. Spatial pattern of mean annual maximum temperature.

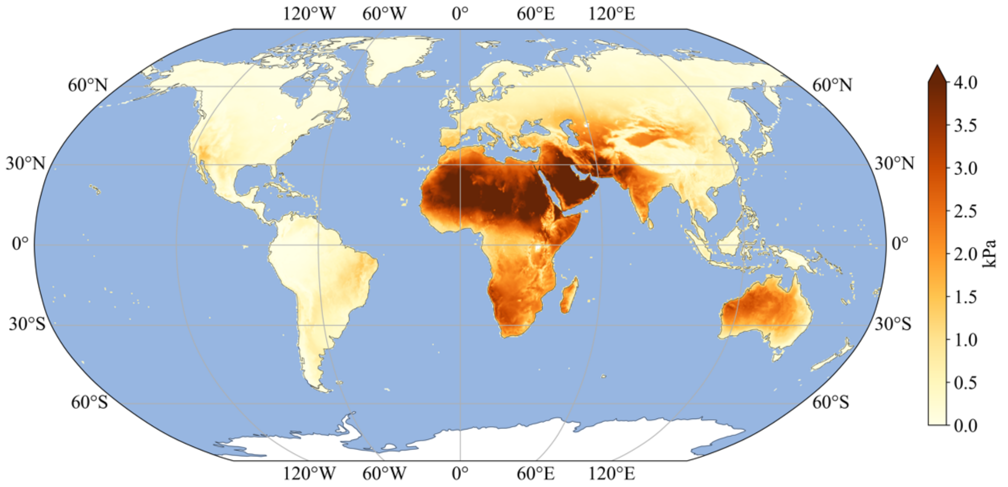

Supplementary Fig 13. Spatial pattern of mean annual VPD. Higher values mean drier conditions.

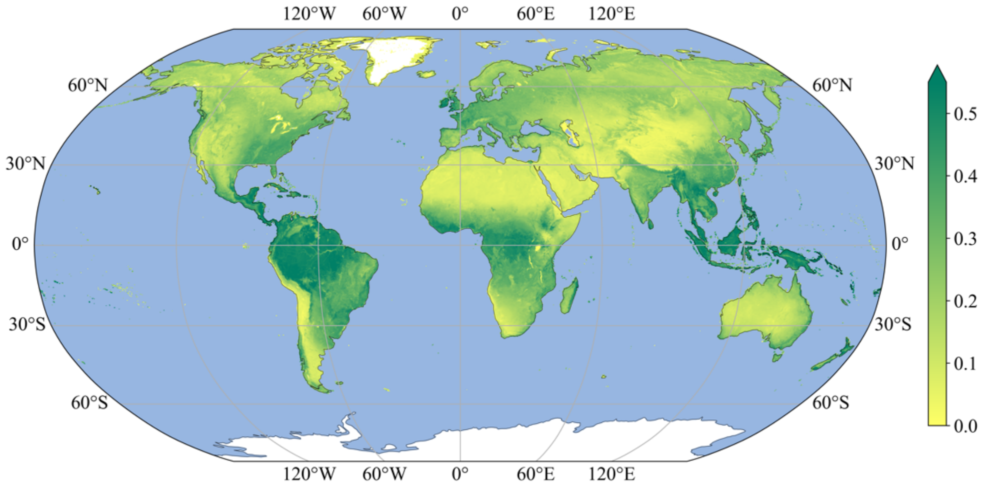

Supplementary Fig 14. Spatial pattern of mean annual EVI.

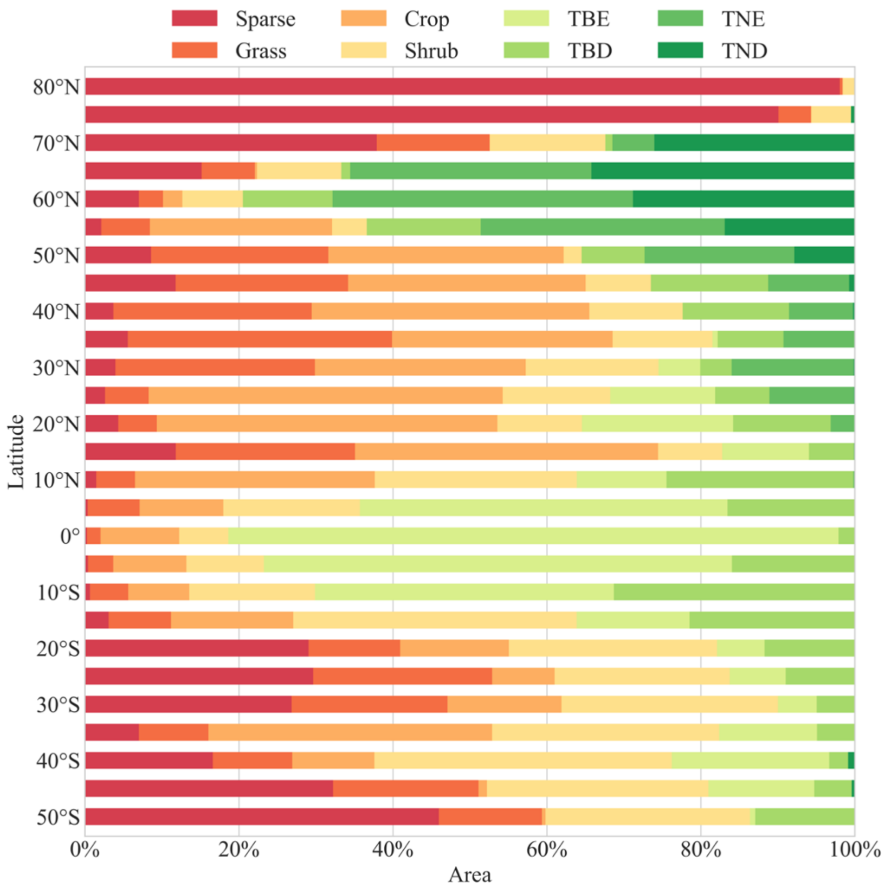

Supplementary Fig 15. Global vegetation distribution with latitude. The vegetation types include sparse vegetation, grassland, cropland, shrubland, tree cover broad-leaf evergreen (TBE), tree cover broad-leaf deciduous (TBD), tree cover needle-leaf evergreen (TNE), tree cover needle-leaf deciduous (TND).

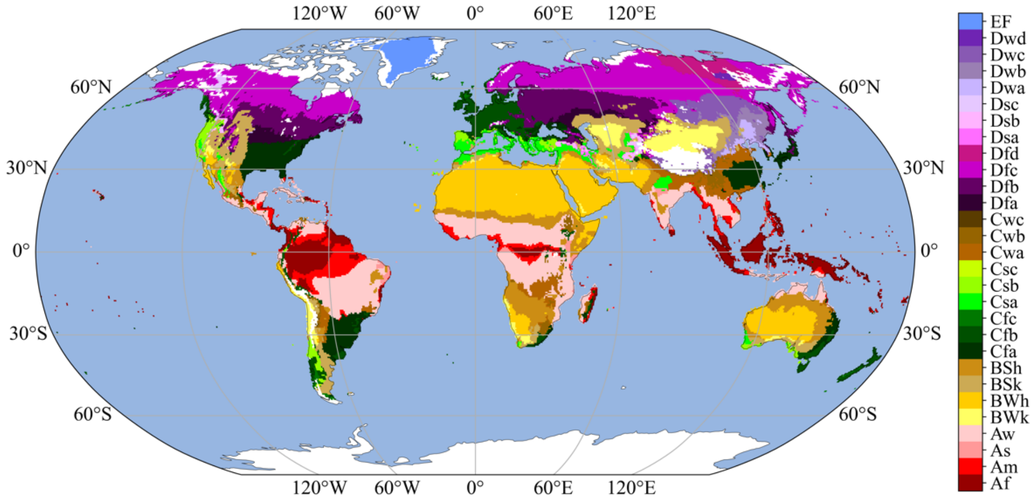

Supplementary Fig 16. Köppen-Geiger climate zone. The main climates include A (equatorial), B (arid), C (warm temperate), D (Snow), and E (polar). The precipitation types include W (desert), S (steppe), f (fully humid), s (summer dry), w (winter dry), and m (monsoonal). The temperature types include h (hot arid), k (cold arid), a (hot summer), b (warm summer), c (cool summer), d (extremely continental), F (polar frost), and T (polar tundra).

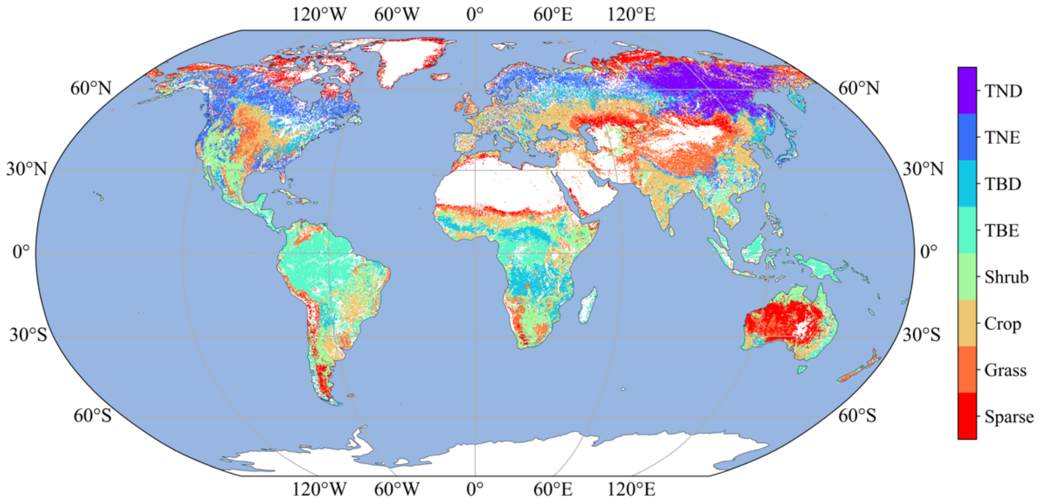

Supplementary Fig 17. Vegetation type from the European Space Agency (ESA) Climate Change Initiative (CCI). The vegetation types include sparse vegetation, grassland, cropland, shrubland, tree cover broad-leaf evergreen (TBE), tree cover broad-leaf deciduous (TBD), tree cover needle-leaf evergreen (TNE), tree cover needle-leaf deciduous (TND).

Supplementary References

1. Montzka C, Herbst M, Weihermüller L, Verhoef A, Vereecken H. A global data set of soil hydraulic properties and sub-grid variability of soil water retention and hydraulic conductivity curves. Earth System Science Data 2017, 9(2): 529-543.
