# Supplementary figures and images for: Wildfire precursors show complementary predictability in different timescales
Source: Nat Commun. 2023 Oct 26;14:6829. doi: 10.1038/s41467-023-42597-5 (PMC10603132; doi:10.1038/s41467-023-42597-5)

**a**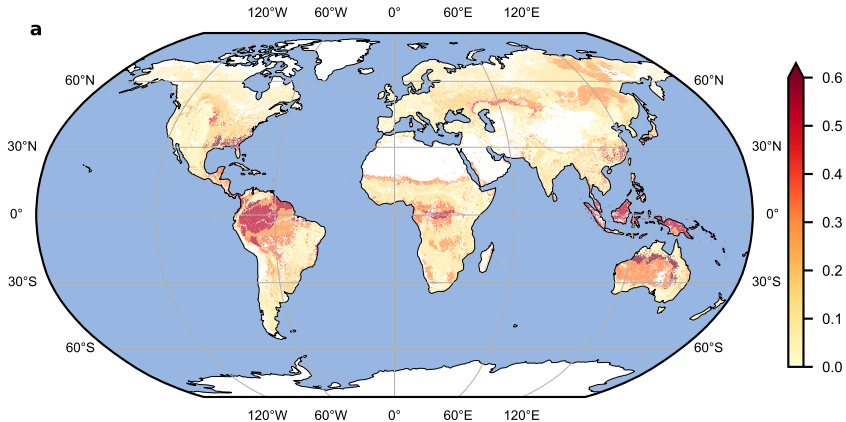

Supplement: Supplementary file 3 — Source Data [file 41467_2023_42597_MOESM3_ESM.zip › Source Data/Source Data Fig1/Fig1a.pdf]

**c**

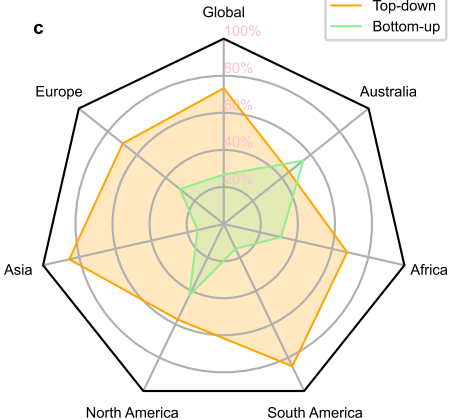

Supplement: Supplementary file 3 — Source Data [file 41467_2023_42597_MOESM3_ESM.zip › Source Data/Source Data Fig1/Fig1c.pdf]

**b**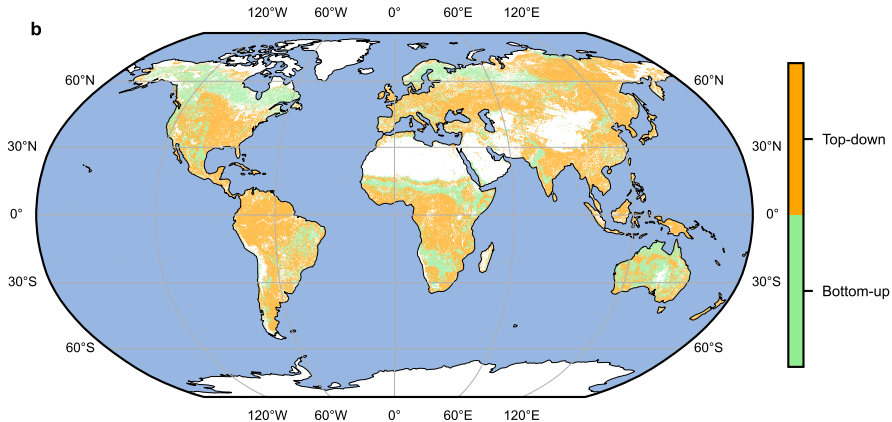

Supplement: Supplementary file 3 — Source Data [file 41467_2023_42597_MOESM3_ESM.zip › Source Data/Source Data Fig1/Fig1b.pdf]

**d**

Negative:

8.4%

78.9%

Positive:

91.6%

21.1%

Density

4

2

-0.6

-0.4

-0.2

0.0

0.2

0.4

0.6

Partial Correlation

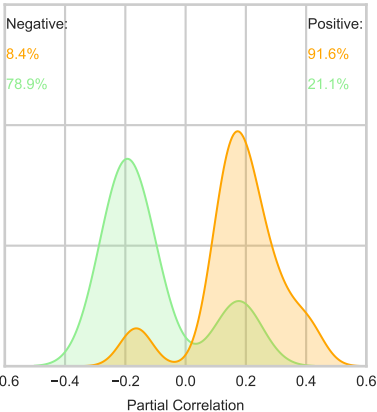

Supplement: Supplementary file 3 — Source Data [file 41467_2023_42597_MOESM3_ESM.zip › Source Data/Source Data Fig1/Fig1d.pdf]

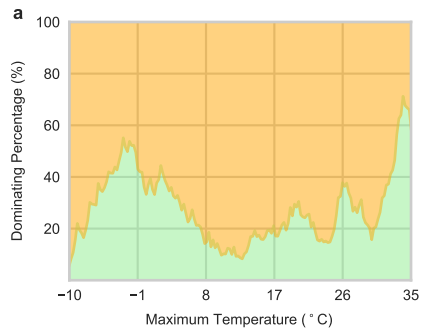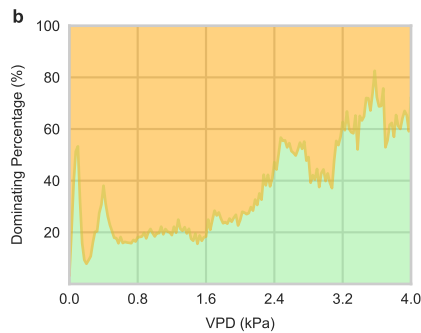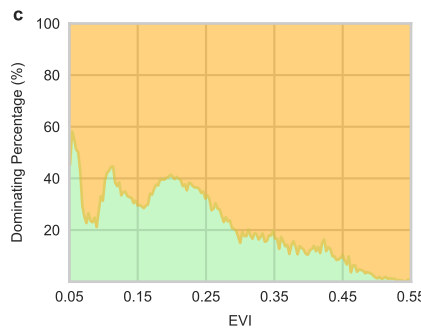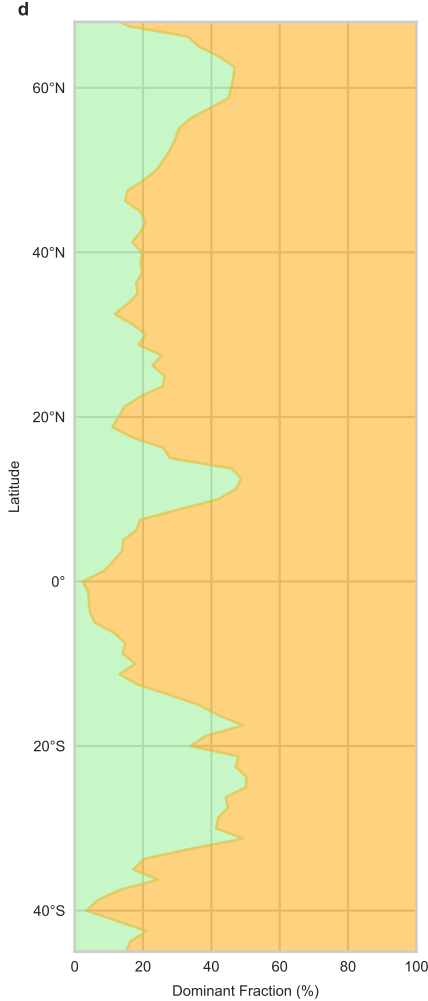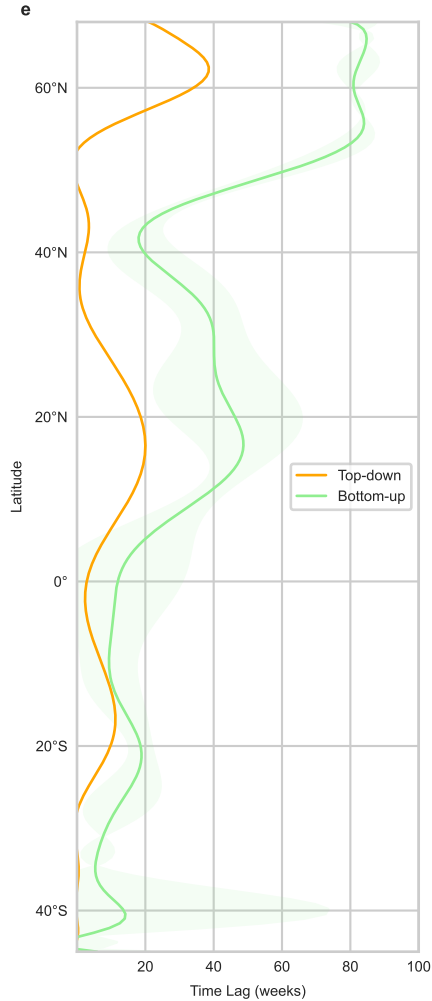

Supplement: Supplementary file 3 — Source Data [file 41467_2023_42597_MOESM3_ESM.zip › Source Data/Source Data Fig3/Fig3.pdf]

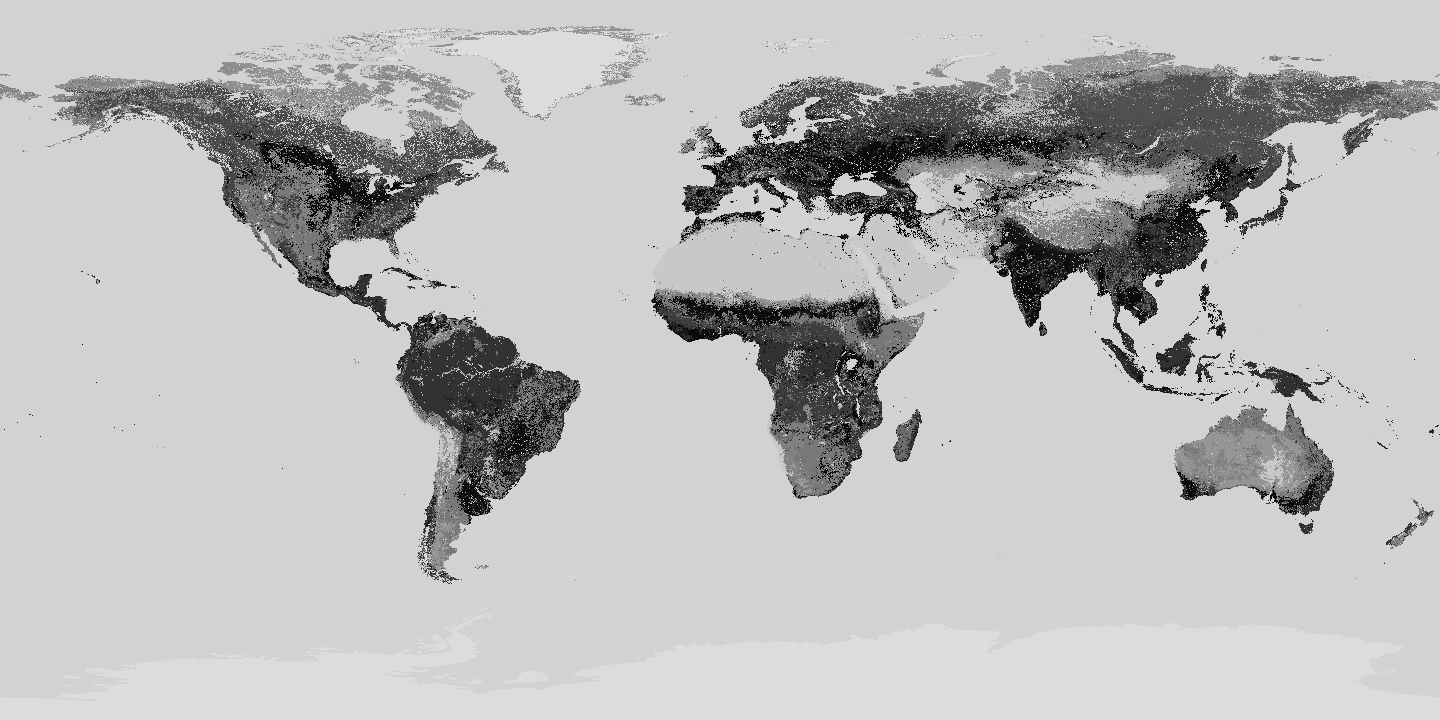

Supplement: Supplementary file 3 — Source Data [file 41467_2023_42597_MOESM3_ESM.zip › Source Data/Source Data Fig4/Land_Cover_025d.tif]

**a**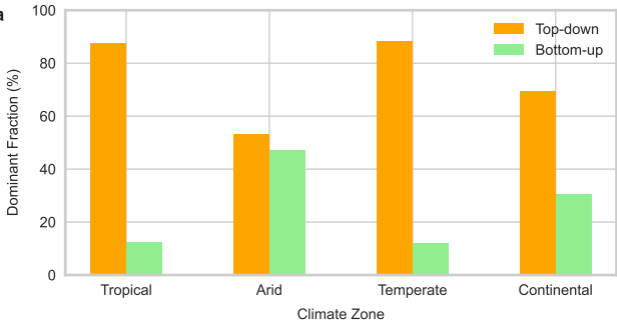

Supplement: Supplementary file 3 — Source Data [file 41467_2023_42597_MOESM3_ESM.zip › Source Data/Source Data Fig4/Fig4a.pdf]

**b**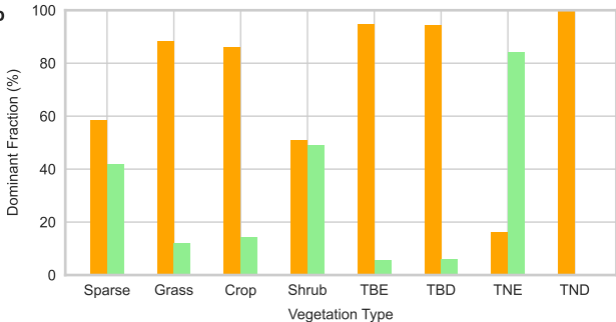

Supplement: Supplementary file 3 — Source Data [file 41467_2023_42597_MOESM3_ESM.zip › Source Data/Source Data Fig4/Fig4b.pdf]

**c**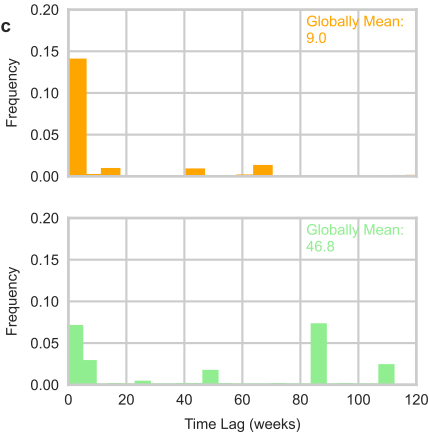

Supplement: Supplementary file 3 — Source Data [file 41467_2023_42597_MOESM3_ESM.zip › Source Data/Source Data Fig2/Fig2c.pdf]

**b**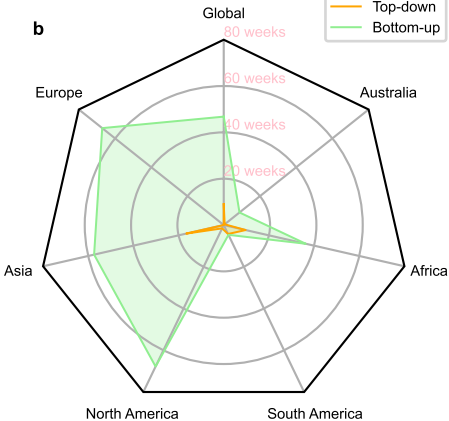

Supplement: Supplementary file 3 — Source Data [file 41467_2023_42597_MOESM3_ESM.zip › Source Data/Source Data Fig2/Fig2b.pdf]

**a**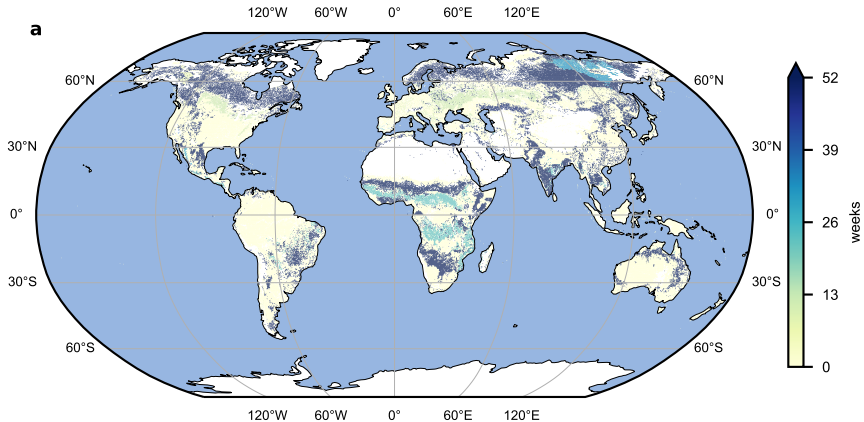

Supplement: Supplementary file 3 — Source Data [file 41467_2023_42597_MOESM3_ESM.zip › Source Data/Source Data Fig2/Fig2a.pdf]
